# Software updates in the Illumina HiSeq platform affect whole-genome bisulfite sequencing

**DOI:** 10.1186/s12864-016-3392-9

**Published:** 2017-01-05

**Authors:** Hidehiro Toh, Kenjiro Shirane, Fumihito Miura, Naoki Kubo, Kenji Ichiyanagi, Katsuhiko Hayashi, Mitinori Saitou, Mikita Suyama, Takashi Ito, Hiroyuki Sasaki

**Affiliations:** 1 Division of Epigenomics and Development, Medical Institute of Bioregulation, Kyushu University, Fukuoka, Japan; 2 Department of Biochemistry, Kyushu University Graduate School of Medical Sciences, Fukuoka, Japan; 3 Department of Stem Cell Biology and Medicine, Kyushu University Graduate School of Medical Sciences, Fukuoka, Japan; 4 Department of Anatomy and Cell Biology, Graduate School of Medicine, Kyoto University, Kyoto, Japan; 5 Division of Bioinformatics, Medical Institute of Bioregulation, Kyushu University, Fukuoka, Japan

**Keywords:** Whole-genome bisulfite sequencing, DNA methylation, Illumina HiSeq platform, HiSeq control software

## Abstract

**Background:**

Methylation of cytosine in genomic DNA is a well-characterized epigenetic modification involved in many cellular processes and diseases. Whole-genome bisulfite sequencing (WGBS), such as MethylC-seq and post-bisulfite adaptor tagging sequencing (PBAT-seq), uses the power of high-throughput DNA sequencers and provides genome-wide DNA methylation profiles at single-base resolution. However, the accuracy and consistency of WGBS outputs in relation to the operating conditions of high-throughput sequencers have not been explored.

**Results:**

We have used the Illumina HiSeq platform for our PBAT-based WGBS, and found that different versions of HiSeq Control Software (HCS) and Real-Time Analysis (RTA) installed on the system provided different global CpG methylation levels (approximately 5% overall difference) for the same libraries. This problem was reproduced multiple times with different WGBS libraries and likely to be associated with the low sequence diversity of bisulfite-converted DNA. We found that HCS was the major determinant in the observed differences. To determine which version of HCS is most suitable for WGBS, we used substrates with predetermined CpG methylation levels, and found that HCS v2.0.5 is the best among the examined versions. HCS v2.0.12 showed the poorest performance and provided artificially lower CpG methylation levels when 5-methylcytosine is read as guanine (first read of PBAT-seq and second read of MethylC-seq). In addition, paired-end sequencing of low diversity libraries using HCS v2.2.38 or the latest HCS v2.2.58 was greatly affected by cluster densities.

**Conclusions:**

Software updates in the Illumina HiSeq platform can affect the outputs from low-diversity sequencing libraries such as WGBS libraries. More recent versions are not necessarily the better, and HCS v2.0.5 is currently the best for WGBS among the examined HCS versions. Thus, together with other experimental conditions, special care has to be taken on this point when CpG methylation levels are to be compared between different samples by WGBS.

**Electronic supplementary material:**

The online version of this article (doi:10.1186/s12864-016-3392-9) contains supplementary material, which is available to authorized users.

## Background

Methylation of cytosine (C) in genomic DNA is a well-characterized epigenetic modification involved in many cellular processes, including differentiation, genomic imprinting, X-chromosome inactivation, transposon silencing, chromosome stability, and maintenance of homeostasis. Aberrant DNA methylation has been reported in a growing number of human diseases, such as cancer, developmental diseases, and metabolic disorders [1]. Until a decade ago, DNA methylation studies only focused on small regions of the genome because of technical limitations. However, recent advances in DNA sequencing technology has made it possible to construct single-base resolution maps of 5-methylcytosine (5mC) at the genome-wide scale [2]. The technology is collectively called whole-genome bisulfite sequencing (WGBS) or methylome analysis, and its practical methods include MethylC-seq [3] and post-bisulfite adaptor tagging sequencing (PBAT-seq) [4]. Reduced representation bisulfite sequencing (RRBS) is also used for single-base resolution 5mC mapping in CpG-rich regions of the genome [5]. The first complete methylome maps were constructed by MethylC-seq [6–8], and then the PBAT method was developed for performing amplification-free WGBS of a nanogram quantity of DNA [4]. With this method, methylome maps were constructed for human and mouse cells [9–15] as well as plant and fungal cells [4, 16]. WGBS is increasingly important in biology and medicine and the International Human Epigenome Consortium (IHEC) recommends WGBS as the standard method for DNA methylation analysis (http://ihec-epigenomes.org/).

The Illumina HiSeq platform accounts for the majority of WGBS studies that are currently under way because this technology generates the largest amount of data per run at the lowest cost per base among the high-throughput sequencers [2, 17]. Like many other laboratories, we have been using the HiSeq platform for our PBAT-based WGBS. In the base calling system of HiSeq, the HiSeq Control Software (HCS) locates clusters, extracts intensity, and calculates color matrix before the Real-Time Analysis (RTA) performs base calling and quality scoring (Additional file 1: Figure S1a). Accurate base calling requires sequence diversity because identification of individual clusters and determination of their coordinates by HCS relies on the diversity. Thus, low sequence diversity samples, including bisulfite-converted DNAs, are obviously not the best substrates for HiSeq sequencing [18].

In the course of our WGBS study on mouse spermatogonia [13], we realized that different versions of HCS and RTA installed on the HiSeq system provided different global CpG methylation levels (approximately 5% difference) for the same libraries. This problem was reproduced in our system using different WGBS libraries and also in HiSeq systems of other laboratories. We found that the first read of PBAT-seq and the second read of MethylC-seq were affected. Thus, it appeared that the problem resides in inaccurate calling of guanine (G), which appears at the position corresponding to 5mC in the complementary strand. These and other observations suggest that software updates can affect the sequence outputs from low diversity libraries such as WGBS libraries. Here we describe the details of the problem, determine which versions of HCS and RTA are more reliable, and discuss our recommendations to minimize the problem.

## Results and discussion

### Different HCS and RTA versions provide different CpG methylation levels

WGBS relies on bisulfite conversion of unmethylated C, but not 5mC, to uracil. Because 5mC normally occupies only a small proportion of Cs, bisulfite-treated DNA shows depletion of C, resulting in a low diversity sequence. PBAT-seq is designed to generate sequence reads complementary to the bisulfite-converted strand, and thus 5mC appears as G in the first read (R1) and as C in the second read (R2) (Additional file 1: Figure S1b) [4]. In the course of our WGBS study of early postnatal mouse spermatogonia [13], we realized that the global CpG methylation level determined by PBAT-seq of the same library significantly changed upon HCS and RTA updates of the HiSeq sequencer. Therefore, we set out to examine the generality of the problem and to explore the causes. Throughout this paper, global CpG methylation levels refer to weighted levels, which take sequencing depth into account [19].

**Fig. 1:**
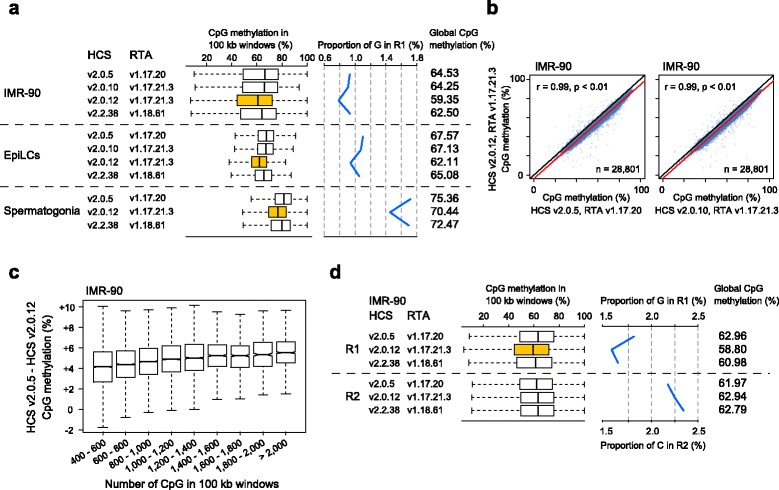
Different CpG methylation levels obtained from identical PBAT libraries using different HCS and RTA versions. **a** CpG methylation levels determined by single-end PBAT-seq. CpG methylation levels in 100 kb windows are shown as a box plot (*left*). Different proportions of G among the four bases in R1 obtained using different HCS and RTA versions are shown as a line plot (*right*). **b** Correlation between the CpG methylation levels determined using HCS v2.0.5, v2.0.10, and v2.0.12. CpG methylation values of 100-kb non-overlapping sliding windows across the autosomes are plotted with a linear regression line (*red*). **c** Differences between the CpG methylation levels determined using HCS v2.0.5 and v2.0.12 against the CpG density. CpG methylation values were calculated in 100-kb non-overlapping sliding windows across the autosomes. All 100 kb windows were grouped into nine classes according to the number of contained CpG. **d** CpG methylation levels determined by paired-end PBAT-seq (IMR-90). CpG methylation levels in 100 kb windows are shown as a box plot (*left*). Different proportions of G in R1 and C in R2 obtained using different HCS and RTA versions are shown as a line plot (*right*)

We performed a series of single-end runs using three PBAT libraries (IMR-90 human fibroblasts, mouse epiblast-like cells [EpiLCs], and mouse spermatogonia). Each library was sequenced multiple times (replicates) and each replicate run was performed using a lane of flow cell on a HiSeq 1500 or HiSeq 2500 sequencers (Additional file 1: Figure S1c). We mapped single-end sequence reads trimmed to 96 bases on the human (hg19) or the mouse (mm10) reference genome and obtained 59–99 million uniquely mapped reads per lane (Additional file 1: Table S1). We confirmed that different combinations of HCS and RTA versions provided different global CpG methylation levels (up to approximately 5% difference) for the same libraries (Fig. 1a, b, Additional file 1: Figure S2a). Such differences were observed even when an identical HiSeq sequencer was used (Additional file 1: Figure S1d). The global CpG methylation difference of 5% was not negligible because similar differences were observed in several types of mouse cells during differentiation (Additional file 1: Figure S3).

For the three libraries, HCS v2.0.5 always provided the highest CpG methylation level, and HCS v2.0.12 the lowest (Fig. 1a). Compared with the other HCS versions, HCS v2.0.12 provided less Gs (Fig. 1a), indicating that a decreased G count is the cause of the lower methylation levels. HCS v2.0.10 provided a methylation level approximately 5% higher than that obtained by HCS v2.0.12, even though the same RTA version (v1.17.21.3) was used (Fig. 1b, Additional file 1: Figure S2b). Thus, HCS and not RTA was the major determinant of the observed differences. Regions with higher CpG density tended to show larger differences between HCS v2.0.5 and v2.0.12 (Fig. 1c, Additional file 1: Figure S2c). Furthermore, when we tried to identify partially methylated domains (PMDs) in EpiLCs, the two versions gave very different results (Additional file 1: Figure S2d). The PMDs are observed in several cell types including cancer cells [20] and associated with intermediate levels of methylation, specific histone modifications, nuclear lamina, and gene silencing [21, 22], showing that different HCS versions can impact biological outcomes.

We next prepared a paired-end PBAT library from IMR-90 human fibroblasts and compared the results obtained using the three HCS versions (Additional file 1: Table S1). As mentioned above, 5mC appears as G in R1 and as C in R2 in paired-end PBAT-seq (Additional file 1: Figure S1b). R1 data showed CpG methylation differences with different HCS versions (Fig. 1d), similar to those observed by single-end PBAT-seq (Fig. 1a). Interestingly, R2 data showed smaller differences (<1.0%) (Fig. 1d). Thus, R1 data derived by HCS v2.0.12 produced a significantly lower (approximately 4%) methylation level than the corresponding R2 data (Fig. 1d). Ideally, R1 and R2 data should provide identical methylation levels. Among the HCS versions, v2.0.5 produced the closest R1 and R2 methylation levels (Fig. 1d).

We previously performed paired-end MethylC-seq with human genomic DNA (purchased from Promega) using HCS v2.0.12 and RTA v1.17.21.3 (accession no. DRA002280) [14]. MethylC-seq is designed to read 5mC as C in R1 and as G in R2 [3], which is the opposite of PBAT-seq. The global CpG methylation level determined using R2 data (55.5%) was lower than that determined using R1 data (57.4%) as expected. However, the difference between R1 and R2 methylation levels was relatively small (1.9%) compared with the other paired-end WGBS cases. We then realized that this particular paired-end MethylC-seq library had contained unconverted PhiX DNA at 50% w/w. Thus, the 50% w/w PhiX DNA spike-in may have alleviated the problem, perhaps through increasing the sequence diversity, for this run using HCS v2.0.12. These results suggest that R1 of PBAT-seq and R2 of MethylC-seq provide lower methylation levels than the other reads and that the methylation level is lower when 5mC is read as G or is higher when 5mC is read as C.

### The HCS version suitable for WGBS

**Fig. 2:**
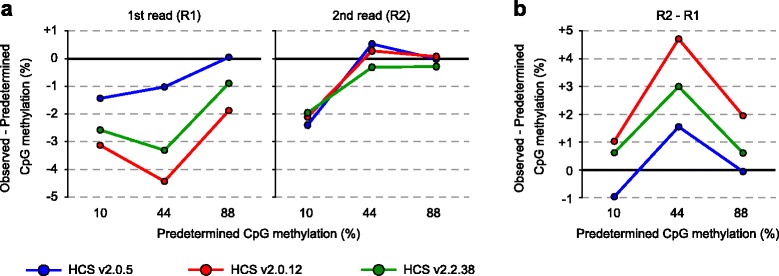
Observed versus predetermined CpG methylation levels of a series of mixture of unmethylated and in vitro methylated lambda DNAs. **a** The differences between the observed and predetermined CpG methylation levels are plotted against the predetermined CpG methylation levels for each HCS version. R1 and R2 data from paired-end PBAT-seq runs were separately analyzed. **b** Differences between the R1 and R2 CpG methylation levels are shown for each HCS version

We then attempted to determine which version of HCS is most suitable for WGBS. To generate substrates with known CpG methylation levels, lambda phage DNA was methylated in vitro to near completion by treatment with *Sss*I methyltransferase. We confirmed the overall resistance of the treated DNA to methylation-sensitive restriction enzyme *Hpa*II (data not shown). Furthermore, we performed bisulfite sequencing at three loci (58 CpG sites), which demonstrated 97.9% CpG methylation (Additional file 1: Figure S4). We prepared a series of lambda DNA mixtures with increasing proportions of the methylated DNA (10, 44, and 88%) and performed paired-end PBAT-seq using the three HCS versions. We mapped the reads onto the lambda phage genome (48,502 base pairs) and obtained 3.5–8.0 million uniquely mapped reads (Additional file 1: Table S2), with the calculated average depths of 3,498–7,962 per strand. R1 data revealed striking differences in CpG methylation between the HCS versions, with HCS v2.0.5 showing the best performance (closest to the predetermined level) and v2.0.12 the poorest (Fig. 2a). In contrast, R2 data showed smaller differences, and all data were close to the predetermined level (Fig. 2a). HCS v2.0.5 provided the least differences between R1 and R2 methylation levels (Fig. 2b), consistent with the findings in human and mouse genomic DNAs (Fig. 1d). HCS v2.0.12 always provided the lowest methylation levels in R1 among the different versions (Figs. 1a, d, and 2a). These results indicate that HCS v2.0.5 is most suitable for WGBS among the three HCS versions and that HCS v2.0.12 provides methylation levels lower than the real values when 5mC is read as G. Since R2 data obtained with different HCS versions were all close to the real values, single-end MethylC-seq, where 5mC is read as C, should not be affected by the versions.

### Quality scores assigned to 5mCs

**Fig. 3:**
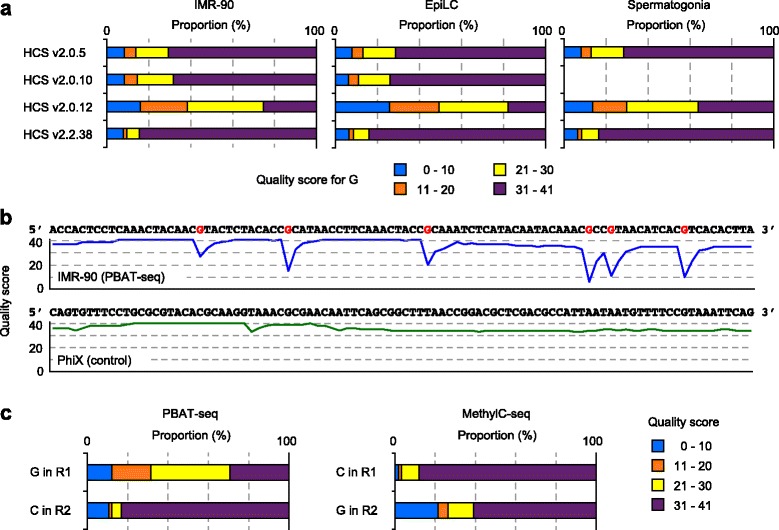
Quality scores assigned to base G. **a** Quality scores assigned to Gs in the raw reads obtained using different HCS versions. All Gs were grouped into four classes according to the assigned quality score. **b** Examples of drops in quality score at Gs. Representative sequence reads from the PBAT-seq (IMR-90) and control (PhiX) data generated using HCS v2.0.12 are shown. Gs in the IMR-90 read are shown in red. **c** Quality scores assigned to the base representing 5mC (G or C) in R1 and R2 of the paired-end PBAT-seq and MethylC-seq using HCS v2.0.12. In the MethylC-seq, 50% w/w PhiX DNA was spiked in (accession no. DRA002280) [14]

Next, we examined the quality scores assigned to the respective bases of the PBAT-seq reads. In the single-end PBAT-seq data obtained from IMR-90 human fibroblasts, mouse EpiLCs, and mouse spermatogonia, quality scores over 30 (99.9% accuracy) were assigned to over 85% of the bases other than G (Additional file 1: Figure S5). However, the quality scores assigned to G greatly changed depending on the HCS versions. In particular, HCS v2.0.12 assigned low quality scores to G (only 18–36% of G had quality scores over 30) (Fig. 3a). Steep drops (>10) in quality score were observed at Gs in 78.0% of the sequence reads containing at least one G (IMR-90) (Fig. 3b). In contrast, high quality scores were consistently observed at Gs in the unconverted PhiX phage control lane of the same flow cell (Fig. 3b). HCS v2.0.10 assigned better quality scores to Gs than HCS v2.0.12 with the same RTA version (v1.17.21.3) (Fig. 3a). We did not find low score assignments to Gs in our PBAT-seq data generated using earlier HCS versions, including HCS v1.5.15, v1.4.8, and v1.1.37 (data not shown), suggesting that HCS v1 may not have the problem in G calling.

In the paired-end PBAT-seq using HCS v2.0.12 (IMR-90), Gs in R1 showed lower quality scores than Cs in R2 (Fig. 3c). In contrast, in the paired-end MethylC-seq using HCS v2.0.12 (accession no. DRA002280) [14], Gs in R2 had lower quality scores than Cs in R1, even though PhiX DNA was added at 50% w/w to confer sufficient sequence diversity (Fig. 3c). These results showed that HCS v2.0.12 has problems in scoring the fewest G bases in low diversity samples showing depletion of Gs. The low quality scores and fewer G outputs may be linked to each other, and both are likely due to the fact that base G has the lowest fluorescence intensity among the four bases [23].

### Effect of cluster density on WGBS

The identification of individual clusters and determination of their coordinates by HCS relies on sequence diversity because discrimination of clusters in close proximity requires different fluorescence signals in the initial cycles. Thus, it has been reported that low diversity sequencing is affected in a high cluster density range [18]. To investigate the relationships between cluster density, HCS version, and CpG methylation level in WGBS, we prepared a series of dilutions of the paired-end PBAT library (IMR-90), loaded them onto flow cell lanes, and created different cluster densities (323–629 K per mm^2^; recommended range for v3 cluster kits 750–850 K per mm^2^) (Additional file 1: Table S3). The three HCS versions provided similar CpG methylation levels (<0.5% difference) at different cluster densities (Additional file 1: Table S3), indicating that lower cluster densities have little impact in this case.

**Fig. 4:**
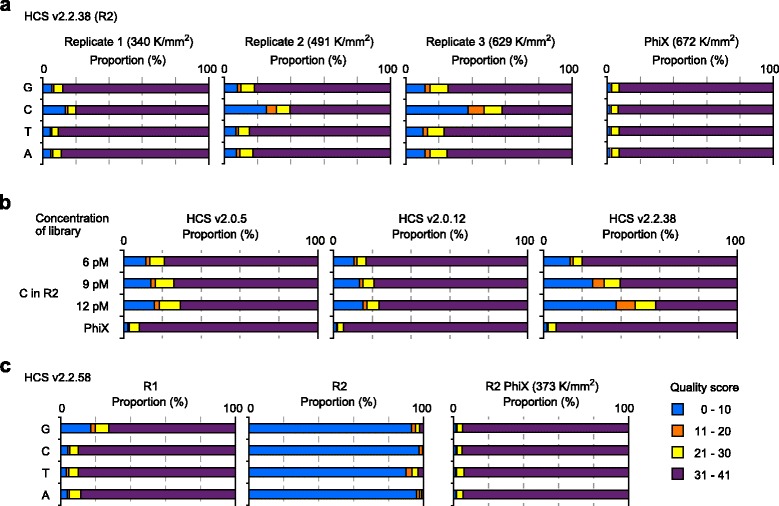
Effect of cluster density on paired-end PBAT-seq (IMR-90). **a** Quality scores assigned to the four bases in R2 generated using HCS v2.2.38 at different cluster densities. The quality scores in R2 of PhiX control on the same flow cell are also shown. **b** Quality scores assigned to Cs in R2 generated using different HCS versions at different cluster densities. **c** Quality scores assigned to the four bases in R1 and R2 generated using the latest HCS v2.2.58 at a modest cluster density (483 K per mm^2^). The quality scores in R2 of PhiX control on the same flow cell are also shown

However, HCS v2.2.38 assigned lower quality scores to Cs in R2, as the cluster density increases (Fig. 4a). Such a density-dependent decrease in quality score for C was not observed with the other HCS versions (v2.0.5 and v.2.0.12) (Fig. 4b). Also, Gs of R1 of the same sequencing run did not show such drops in quality score (data not shown). Because the PhiX control at a high cluster density (672 K per mm^2^) showed good quality scores at Cs in R2 (Fig. 4a), we speculate that HCS v2.2.38 provides lower quality scores to the fewest bases in low-diversity R2 data. Furthermore, R2 data generated using HCS v2.2.38 at 629 K per mm^2^ provided a high global non-CpG (CpA, CpT, and CpC) methylation level (1.86%) for IMR-90 human fibroblasts, which is clearly different from other data (<0.1%) [24], suggesting that R2 data may be less accurate.

Recently, HCS v2.2.58 and RTA v1.18.64 were released from Illumina. To examine the performance of the latest versions, we performed paired-end PBAT-seq on an IMR-90 library constructed from the same DNA as the above studies (Additional file 1: Table S3). These versions provided a global CpG methylation level similar to that obtained by HCS v2.0.5 (62.4% versus 63.0%) in R1. However, they assigned very low quality scores to overall R2 data at a modest cluster density (483 K per mm^2^) (Fig. 4c) and quality scores over 30 were assigned to only 1.8% of all bases.

### WGBS data generated by new Illumina systems

Finally, we analyzed published paired-end WGBS data generated by new Illumina systems, HiSeq 4000 and NextSeq 500. HCS v3.3.x is installed on HiSeq 4000, which uses patterned flow cell technology. We analyzed MethylC-seq data (GSM1707686 and GSM2137773) generated by HiSeq 4000 and found that the difference between R1 and R2 global CpG methylation levels was 2.3% in both data sets. As expected, R1 produced a higher methylation level than corresponding R2 (Additional file 1: Figure S6a). Quality scores over 30 were assigned to approximately 50% of the Gs in R2 (Additional file 1: Figure S6a). Thus, the performance of HiSeq 4000 seemed better than HCS v.2.0.12, but it was not clear whether its performance was better than HCS v2.0.5.

We also analyzed MethylC-seq data (GSM1973803 and GSM1973807) generated by NextSeq 500, which uses a two-color chemistry. Calls for G are made where there is actually no signal on a flow cell. In this run, 30% PhiX DNA was spiked in [25]. We found that the difference between R1 and R2 global CpG methylation levels was relatively small (1.9 and 0.6%) (Additional file 1: Figure S6b). Quality scores over 30 were assigned to 73% of the Gs in R2 (Additional file 1: Figure S6b). Taken together, WGBS outputs by HiSeq 4000 and NextSeq 500 produced better results than HCS v2.0.12. Since we had no information on the software versions and cluster densities, further validation requires replicates from the same WGBS libraries.

## Conclusions

In this study, we found the following regarding the use of Illumina HiSeq sequencers for WGBS. (1) HCS v2.0.5 is currently the best HCS version among the HCS v2 for WGBS (both single-end and paired-end). This version provides CpG methylation levels closest to the real values. (2) It is better to avoid using HCS v2.0.12 for WGBS. This version provides methylation levels lower than the real values (up to approximately 5% difference) and assigns very low quality scores to G bases. (3) R2 of paired-end sequencing of low diversity libraries using HCS v2.2.38 or the latest HCS v2.2.58 is greatly affected by cluster densities. Thus, when using HCS v2.2.38 or v2.2.58, it is better to choose single-end sequencing.

Based on these findings, we suggest the following for WGBS using the HiSeq platform. (1) The same HCS version should be used for data comparisons, whenever possible. (2) Avoid using protocols that read 5mC as G, whenever possible. Choose single-end (not paired-end) sequencing for MethylC-seq and RRBS. We are currently developing a single-end PBAT protocol where 5mC can be represented by C. Addition of 50% w/w PhiX DNA may alleviate the problem, but not fully. (3) Check the quality scores of 5mC in each read. Data showing lower quality scores at 5mC appear to be less accurate, even if its overall quality score is high. (4) Describe the versions of HCS and RTA when publishing WGBS results. It is also helpful to provide the information as metadata in databases. This will help the users to judge whether they can be used for comparison.

## Methods

### Biological materials

Mouse genomic DNA (C57BL/6) was isolated as previously described [13]. IMR-90, a human fibroblast cell line, was cultured as previously described [26].

### Methylation of lambda DNA

To generate methylated control DNA, 1 μg of lambda phage DNA (Promega) was methylated with CpG methyltransferase *Sss*I (New England BioLabs) for 3 h at 37 °C. Near complete methylation was confirmed by the resistance to methylation-sensitive restriction enzyme *Hpa*II (New England BioLabs). Then, 100 ng of the DNA was bisulfite converted and three lambda loci were amplified by polymerase chain reaction (PCR) (95 °C for 30 s followed by 15 cycles of 95 °C for 30 s, 61 °C for 30 s, and 72 °C for 30 s). The PCR products were cloned into pMD20 (TaKaRa) and sequenced. This analysis demonstrated a 97.9% CpG methylation level (Additional file 1: Figure S4). The PCR primers used are listed in Additional file 1: Table S4.

### Preparation of the PBAT library

DNAs samples were subjected to bisulfite treatment with the EZ DNA Methylation-Gold Kit (Zymo Research). PBAT libraries were constructed as described [4], using a new second-strand-synthesis primer: 5′-CAA GCA GAA GAC GGC ATA CGA GAT XXX XXX GTA AAA CGA CGG CCA GCA GGA AAC AGC TAT GAC NNN N-3′ (XXX XXX indicate the sample specific Illumina index tag). Concentrations of the PBAT products were quantified using the KAPA Illumina Library Quantification Kit (Kapa Biosystems).

### Illumina HiSeq sequencing and data analysis

The libraries were sequenced on a HiSeq 2500 or a HiSeq 1500 sequencer to generate 101-nt single-end or paired-end reads. Cluster generation and sequencing were performed in a single-read mode using the TruSeq SR/PE Cluster Kit v3-cBot-HS (Illumina) and TruSeq SBS Kit v3-HS (Illumina) according to the manufacturer’s protocols. In all runs, one of the eight lanes (lane 5) on a flow cell was used as a dedicated control lane (PhiX) for matrix and phasing calculations (cluster density 290–717 K per mm^2^). To avoid potential lane-specific effects, we used the same lane for the same library in all paired-end runs. We truncated raw sequence reads to 96 bases to remove the remaining adapter sequences from the 5′ end and one base from the 3′ end. The resulting reads were aligned to the reference human genome (hg19), mouse genome (mm10), or lambda genome (accession no. J02459) using Bismark v0.10.0 [27]. We used parameters of 28 for the seed length, 1 for the maximum number of mismatches permitted in the seed, and the option “--pbat” that works for PBAT libraries. Only uniquely aligned reads were analyzed.

### Identification of PMDs

We used a sliding window approach to find PMDs, as described previously [8]. First, each chromosome is divided into 10 kb non-overlapping windows. When a 10-kb window contained ten or more CpG sites, each of which were covered at least once, the CpG methylation level of the window was calculated. When the CpG methylation level of the 10-kb window was less than 70%, the window was defined as a PMD window. Contiguous PMD windows were collapsed into a single PMD, and then only those longer than 100 kb were picked up in this study.

## Additional file

Additional file 1: Table S1. Summary of PBAT-seq with human and mouse genomic DNA using different HCS and RTA versions. **Table S2.** Summary of PBAT-seq with in vitro methylated lambda phage DNA. **Table S3.** Summary of the effect of cluster density on PBAT-seq using different HCS versions. **Table S4.** Bisulfite PCR primers for lambda DNA. **Figure S1.** Experimental design to investigate the effect of HCS and RTA updates. **Figure S2.** Different CpG methylation levels obtained for identical PBAT libraries using different HCS and RTA versions. **Figure S3.** Changes in global CpG methylation level during mouse cell differentiation. **Figure S4.** Bisulfite sequencing of in vitro CpG methylated lambda DNA at three selected loci. **Figure S5.** Quality scores assigned to the four bases. **Figure S6.** WGBS data generated by new Illumina systems. (PDF 1211 kb)

## Abbreviations

5mC: 5-methylcytosine
EpiLC: Epiblast-like cell
HCS: HiSeq control software
IHEC: International human epigenome consortium
PBAT-seq: Post-bisulfite adaptor tagging sequencing
RRBS: Reduced representation bisulfite sequencing
RTA: Real-time analysis
WGBS: Whole-genome bisulfite sequencing
