# Supplementary material for: Software updates in the Illumina HiSeq platform affect whole-genome bisulfite sequencing
Source: BMC Genomics. 2017 Jan 5;18:31. doi: 10.1186/s12864-016-3392-9 (PMC5217569; doi:10.1186/s12864-016-3392-9)
Supplement: Additional file 1: Table S1. — Summary of PBAT-seq with human and mouse genomic DNA using different HCS and RTA versions. Table S2. Summary of PBAT-seq with in vitro methylated lambda phage DNA. Table S3. Summary of the effect of cluster density on PBAT-seq using different HCS versions. Table S4. Bisulfite PCR primers for lambda DNA. Figure S1. Experimental design to investigate the effect of HCS and RTA updates. Figure S2. Different CpG methylation levels obtained for identical PBAT libraries using different HCS and RTA versions. Figure S3. Changes in global CpG methylation level during mouse cell differentiation. Figure S4. Bisulfite sequencing of in vitro CpG methylated lambda DNA at three selected loci. Figure S5. Quality scores assigned to the four bases. Figure S6. WGBS data generated by new Illumina systems. (PDF 1211 kb) [file 12864_2016_3392_MOESM1_ESM.pdf]

Table S1. Summary of PBAT-seq with human and mouse genomic DNA using different HCS and RTA versions.

Sample	HCS ver.	RTA ver.	Raw sequence reads	Uniquely mapped reads	Global CpG methylation (%)	Cluster density (K per mm^2)
Single-end PBAT-seq						
IMR-90 human fibroblasts						
Replicate 1	2.0.5	1.17.20	147,291,672	88,123,757	64.53	623
Replicate 2	2.0.10	1.17.21.3	145,913,516	88,212,419	64.25	613
Replicate 3	2.0.12	1.17.21.3	159,621,322	94,323,984	59.35	611
Replicate 4	2.2.38	1.18.61	115,502,660	67,771,996	62.50	437
Mouse EpiLCs						
Replicate 1	2.0.5	1.17.20	94,923,045	58,712,416	66.85	323
Replicate 2	2.0.10	1.17.21.3	138,074,081	85,624,363	67.13	562
Replicate 3	2.0.12	1.17.21.3	151,760,571	93,664,450	62.11	651
Replicate 4	2.2.38	1.18.61	161,484,126	97,793,377	65.08	622
Replicate 5	2.0.5	1.17.20	151,672,897	92,685,305	67.57	647
Mouse spermatogonia						
Replicate 1	2.0.5	1.17.20	140,295,353	88,824,306	75.36	583
Replicate 2	2.0.12	1.17.21.3	158,270,112	99,277,094	70.44	607
Replicate 3	2.2.38	1.18.61	141,682,873	88,427,776	72.47	543
Paired-end PBAT-seq						
IMR-90 human fibroblasts						
Replicate 1 R1	2.0.5	1.17.20	78,252,188	39,898,348	62.96	323
Replicate 1 R2	2.0.5	1.17.20	78,252,188	39,206,518	61.97	323
Replicate 2 R1	2.0.12	1.17.21.3	83,078,788	41,921,568	58.80	342
Replicate 2 R2	2.0.12	1.17.21.3	83,078,788	41,524,432	62.94	342
Replicate 3 R1	2.2.38	1.18.61	82,972,729	42,169,779	60.98	340
Replicate 3 R2	2.2.38	1.18.61	82,972,729	41,410,125	62.79	340

Table S2. Summary of PBAT-seq with *in vitro* methylated lambda phage DNA.

			HCS v2.0.5			HCS v2.0.12			HCS v2.2.38		
			Cluster density (570 K per mm^2)			Cluster density (498 K per mm^2)			Cluster density (615 K per mm^2)		
	Lambda DNA mixtures	Predetermined CpG methylation (%)	Observed CpG methylation (%)	Raw sequence reads	Uniquely mapped reads	Observed CpG methylation (%)	Raw sequence reads	Uniquely mapped reads	Observed CpG methylation (%)	Raw sequence reads	Uniquely mapped reads
R1											
	Mixture 1	9.79	8.36	10,353,036	7,103,653	6.67	7,762,331	5,291,822	7.22	11,801,803	8,045,374
	Mixture 2	44.06	43.08	7,313,884	5,278,424	39.67	5,463,767	3,831,830	40.79	8,369,108	5,909,588
	Mixture 3	88.11	88.25	7,391,724	5,220,790	86.32	5,467,577	3,644,007	87.31	8,224,401	5,567,083
R2											
	Mixture 1	9.79	7.40	10,353,036	6,967,020	7.70	7,762,331	5,137,934	7.84	11,801,803	6,956,117
	Mixture 2	44.06	44.63	7,313,884	5,055,209	44.38	5,463,767	3,685,456	43.79	8,369,108	4,989,115
	Mixture 3	88.11	88.19	7,391,724	4,894,883	88.27	5,467,577	3,535,503	87.91	8,224,401	4,692,949

Table S3. Summary of the effect of cluster density on PBAT-seq using different HCS versions.

		HCS v2.0.5				HCS v2.0.12				HCS v2.2.38			
IMR-90	Concentration of library (pM)	Cluster density (K per mm^2)	Global CpG methylation (%)	Raw sequence reads	Uniquely mapped reads	Cluster density (K per mm^2)	Global CpG methylation (%)	Raw sequence reads	Uniquely mapped reads	Cluster density (K per mm^2)	Global CpG methylation (%)	Raw sequence reads	Uniquely mapped reads
R1													
Replicate 1	6	323	62.96	78,252,188	39,898,348	342	58.80	83,078,788	41,921,568	340	60.98	82,972,729	42,169,779
Replicate 2	9	448	63.19	105,762,701	54,081,978	499	58.64	118,257,044	60,059,700	491	61.25	117,418,244	59,862,353
Replicate 3	12	535	63.20	122,751,869	63,213,898	484	58.88	88,367,906	44,999,796	629	61.51	149,730,667	76,685,013
R2													
Replicate 1	6	323	61.97	78,252,188	39,206,518	342	62.94	83,078,788	41,524,432	340	62.79	82,972,729	41,410,125
Replicate 2	9	448	61.74	105,762,701	53,166,186	499	63.08	118,257,044	59,270,457	491	62.72	117,418,244	57,622,129
Replicate 3	12	535	61.58	122,751,869	62,107,528	484	63.16	88,367,906	44,373,578	629	62.43	149,730,667	72,621,521

		HCS v2.2.58			
IMR-90	Concentration of library (pM)	Cluster density (K per mm^2)	Global CpG methylation (%)	Raw sequence reads	Uniquely mapped reads
R1					
Replicate 1	6	483	62.39	126,281,707	45,557,327
Replicate 2	12	701	62.41	177,071,216	63,487,705
Replicate 3	18	861	62.68	209,021,532	74,114,903
R2					
Replicate 1	6	483	-	126,281,707	-
Replicate 2	12	701	-	177,071,216	-
Replicate 3	18	861	-	209,021,532	-

Table S4. Bisulfite PCR primers for lambda DNA.

Position in the lambda genome	Forward primer	Reverse primer	Reference
951–1,278	5'-GTTATTTTAAAATGTTGTTGGGTGTTTAT-3'	5'-CCTATTCAATATCATCATCAAAAACAA-3'	1
2,164–2,418	5'-GGGTGATTTTTATTAAAGGGGTATT-3'	5'-TACACCATCCTCTTCCTACAAACTC-3'	2
21,648–21,986	5'-GTTTTGGTTGGAGTTAGTATGGAATAGTAA-3'	5'-ATTCTAAATACCATTATACAAACCTCACAATA-3'	1

(1) Tomizawa et al. (2011) Development. 138: 811–820.
(2) Ichiyanagi et al. (2013) Nucleic Acids Res. 41: 738–745.

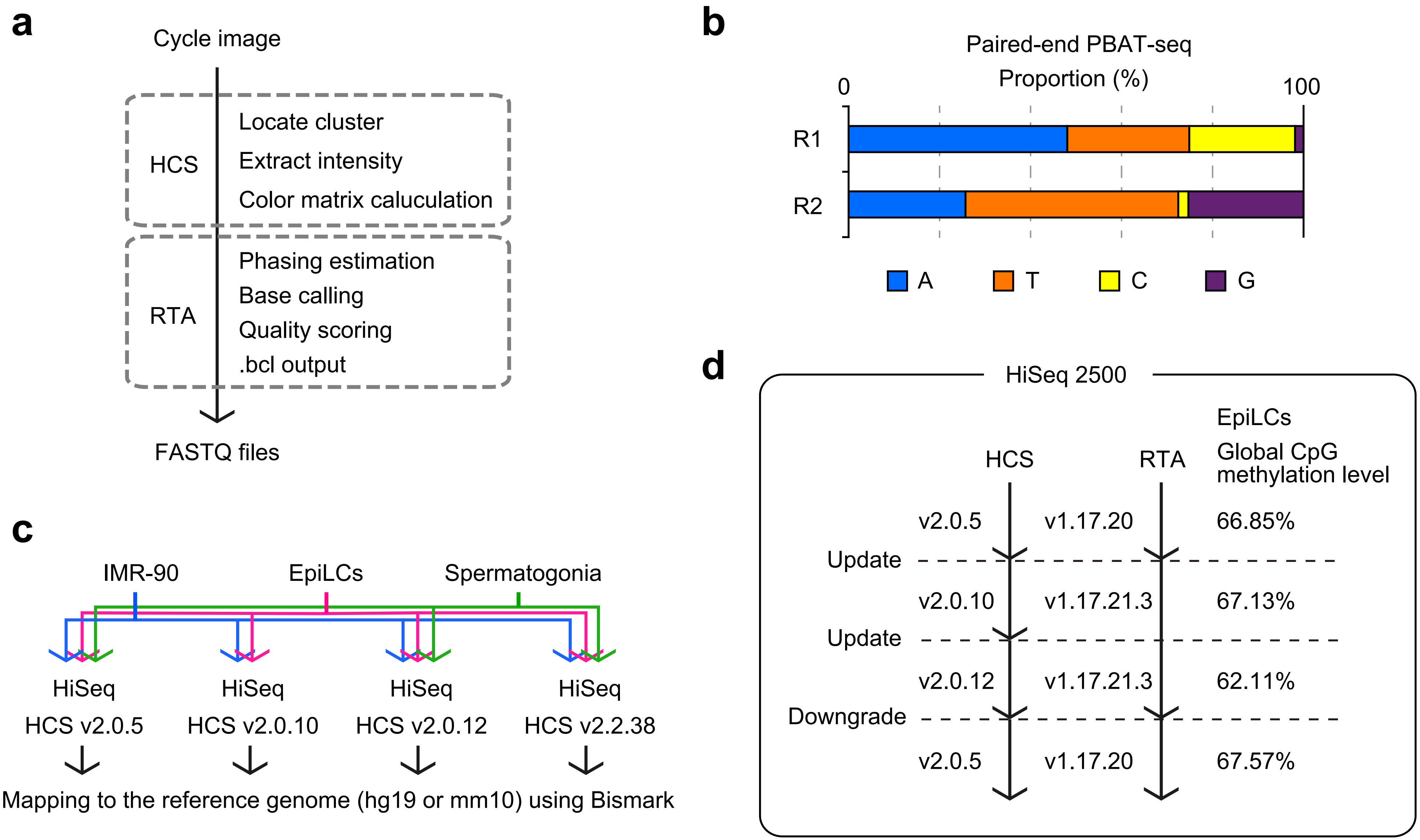

Figure S1. Experimental design to investigate the effect of HCS and RTA updates.

(**a**) Base calling system of the Illumina HiSeq platform. The HCS locates clusters, extracts intensity, and calculates color matrix before the RTA performs base calling and quality scoring. (**b**) Example of the base composition of R1 and R2 of paired-end PBAT-seq. The PBAT-seq is designed to generate sequence reads complementary to the bisulfite-converted strand, and thus 5mC appears as G in R1 and as C in R2. (**c**) Experimental design using single-end PBAT-seq. Each library was sequenced using HiSeq sequencers equipped with four different HCS versions (v2.0.5, v2.0.10, v2.0.12, and v2.2.38). The trimmed raw sequence reads (96 nt) were then mapped to the human or mouse reference genome.
(**d**) Global CpG methylation levels obtained by different HCS and RTA combinations installed in an identical HiSeq sequencer.

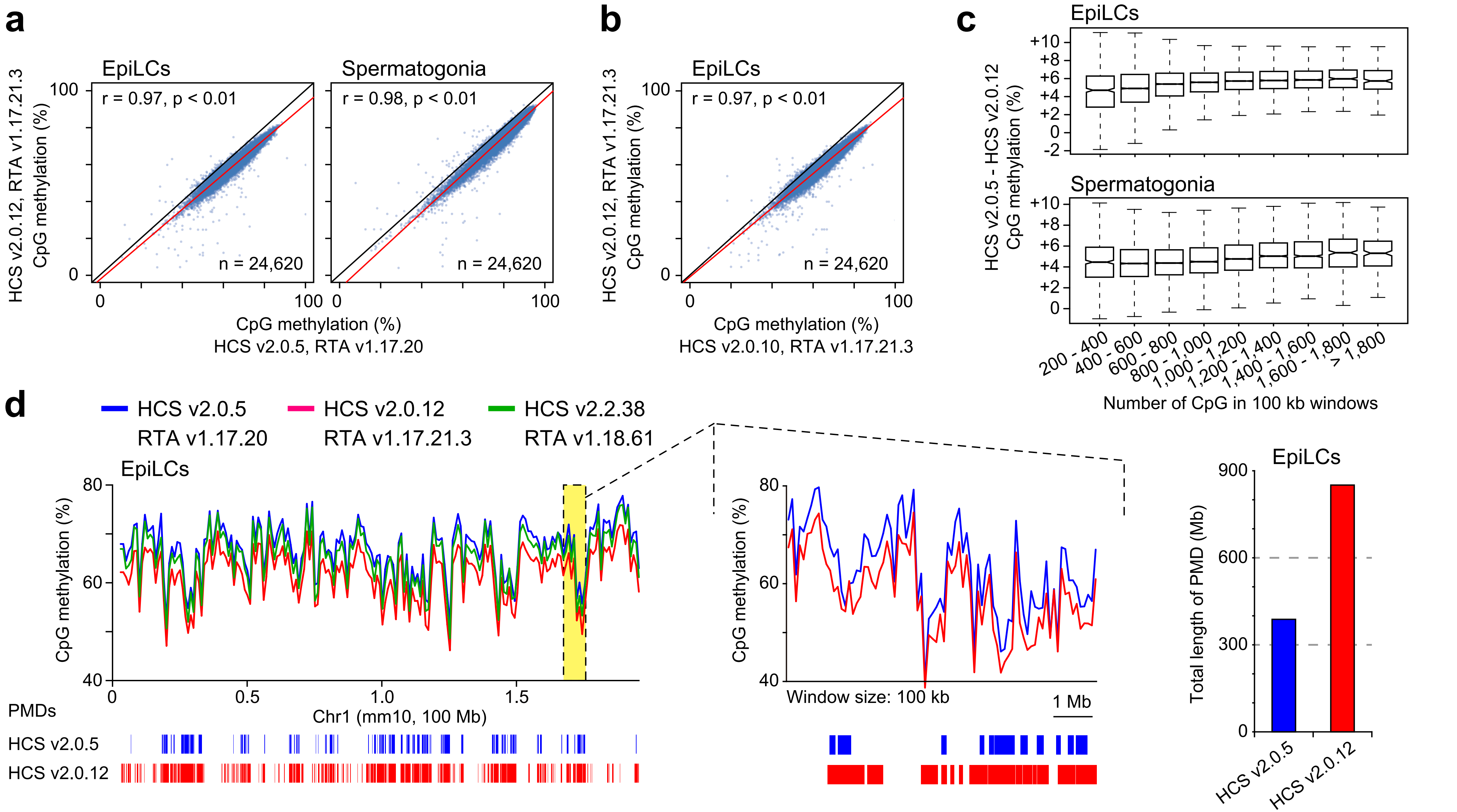

Figure S2. Different CpG methylation levels obtained for identical PBAT libraries using different HCS and RTA versions.

(**a**, **b**) Correlations between the CpG methylation levels determined using HCS v2.0.5 and v2.0.12 (**a**) and HCS v2.0.10 and v2.0.12 (**b**). CpG methylation values of 100-kb non-overlapping sliding windows across the autosomes are plotted with a linear regression line (red). (**c**) CpG methylation difference between the data obtained by HCS v2.0.5 and v2.0.12 plotted against CpG density. CpG methylation values were calculated in non-overlapping sliding windows of 100 kb across the autosomes. All 100 kb windows were grouped into nine classes according to the number of contained CpG. (**d**) CpG methylation profile of mouse EpiLCs across chromosome 1. CpG methylation levels of 1-megabase (Mb) non-overlapping sliding windows determined using HCS v2.0.5, v2.0.12, and v2.2.38 are plotted. PMDs identified using data generated by different versions are shown by blue (HCS v2.0.5) and red rectangles (HCS v2.0.12). A zoom-in view of the indicated region is shown (middle). The total length of PMDs identified using the two HCS versions are also shown (right).

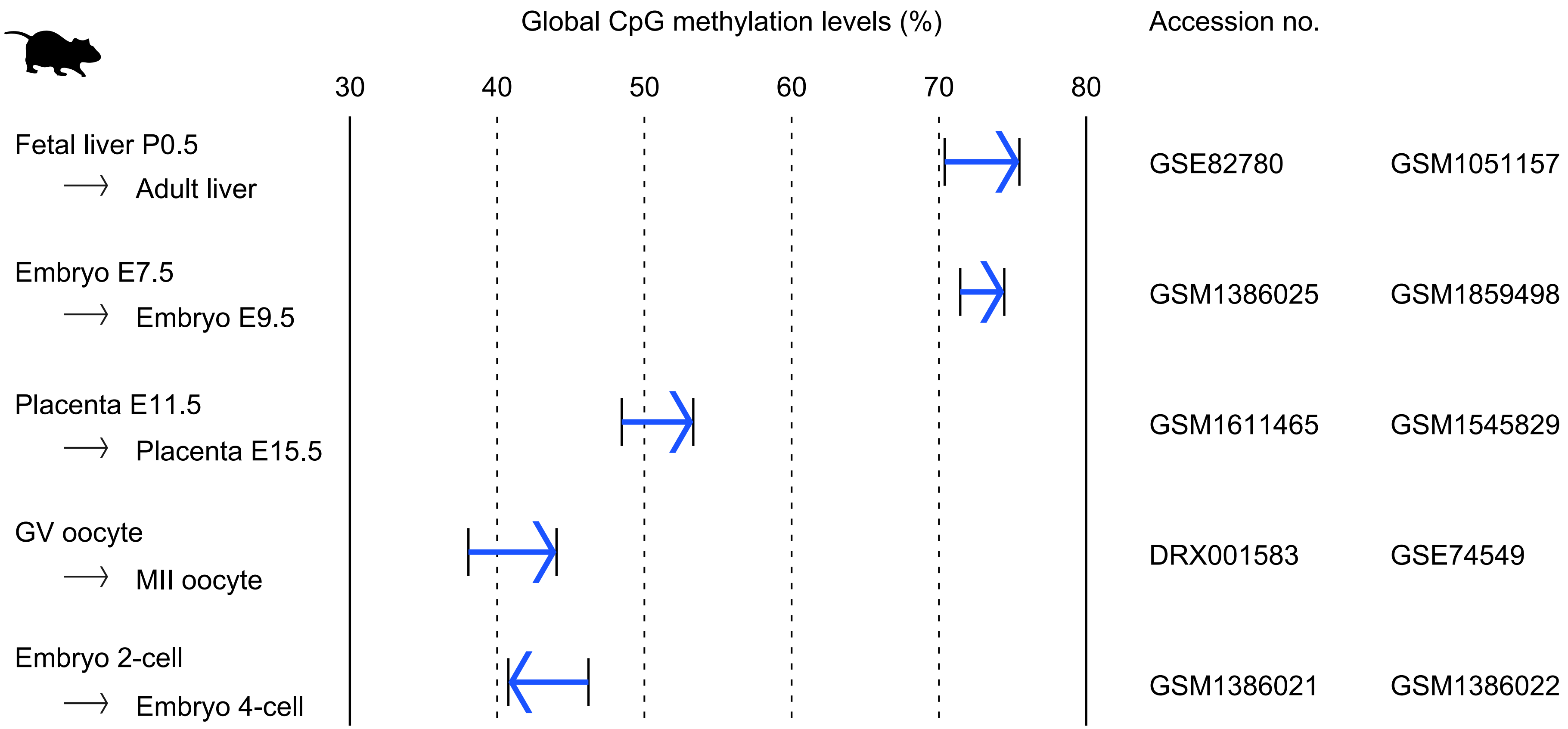

Figure S3. Changes in global CpG methylation level during mouse cell differentiation.

We used publicly available mouse WGBS data sets to compare the global CpG methylation levels during cell differentiation. Raw sequence reads were downloaded and trimmed to remove low-quality bases at the 3′ end, and the resulting reads were aligned to the mouse reference genomes using Bismark v0.10.0. Arrows indicate the changes in global CpG methylation level between the two types of cells. GV oocyte, germinal vesicle oocyte; MII oocyte, metaphase II oocyte.

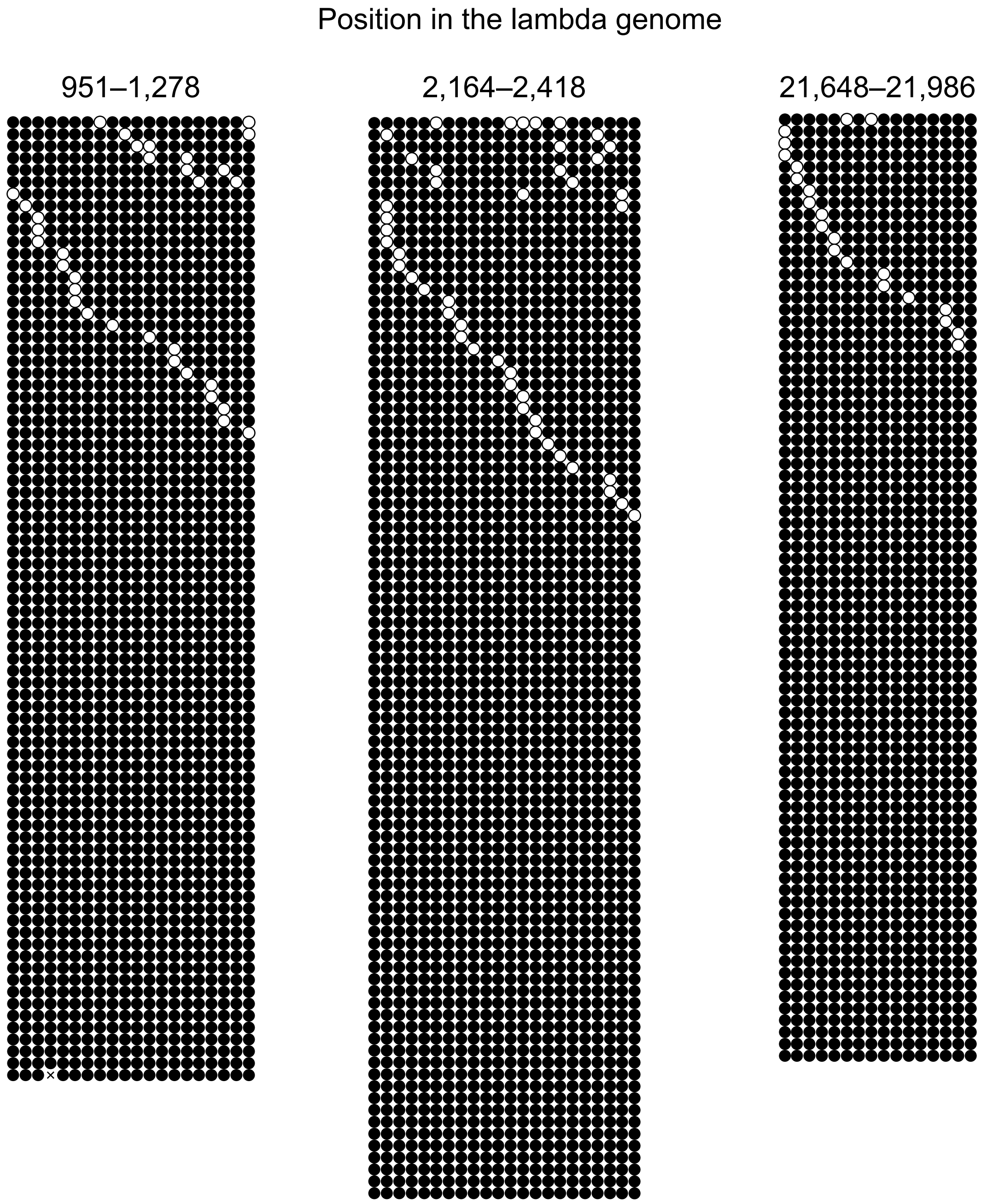

Figure S4. Bisulfite sequencing of *in vitro* CpG methylated lambda DNA at three selected loci.

Methylated and unmethylated CpG sites are shown in filled and open circles, respectively. The PCR primers are shown in Additional file 1: Table S4.

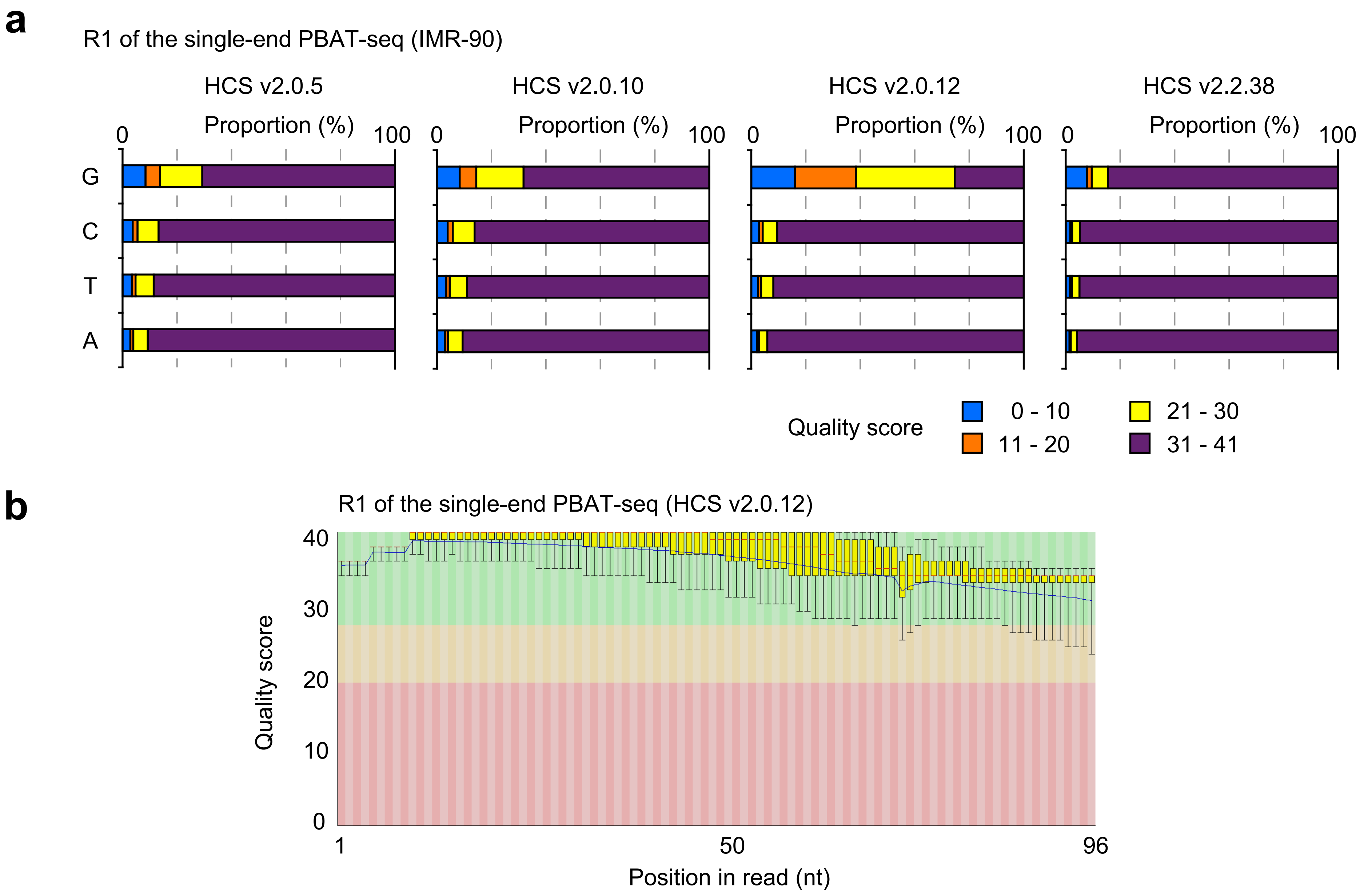

Figure S5. Quality scores assigned to the four bases.

(**a**) Quality scores assigned to the four bases in raw reads obtained using different HCS versions. The quality scores for R1 of the single-end PBAT-seq (IMR-90) are shown. (**b**) Distribution of quality score across the raw reads (IMR-90) generated by FastQC using HCS v2.0.12. This run appeared to generate high-quality sequencing data, but the quality scores assigned to G were low, as shown in (**a**) and Fig. 3b.

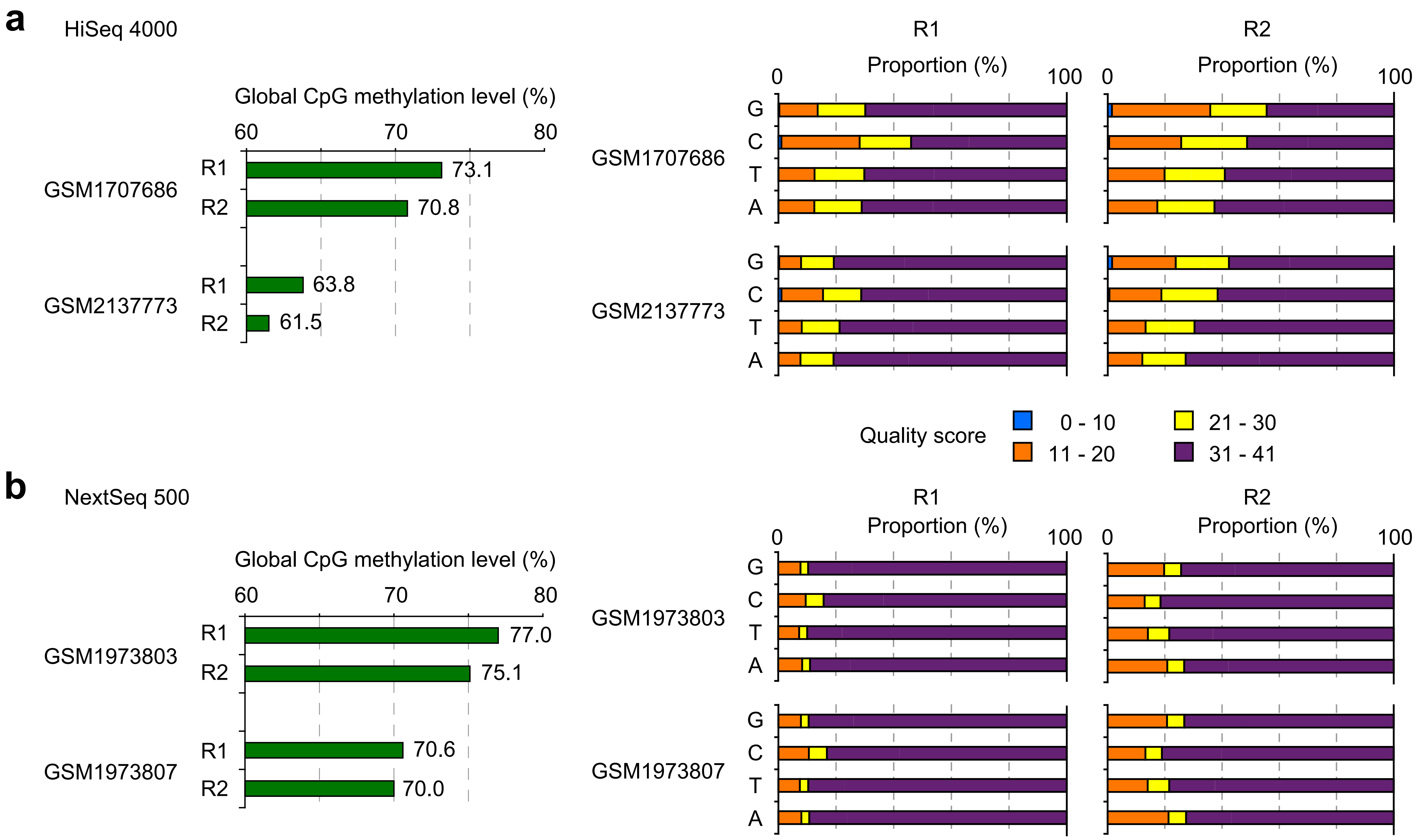

Figure S6. WGBS data generated by new Illumina systems.

(**a**, **b**) Global CpG methylation level and quality scores assigned to the four bases in published paired-end raw sequence reads generated by HiSeq 4000 (**a**) and NextSeq 500 (**b**).
